# Sintilimab plus bevacizumab and CapeOx (BBCAPX) on first-line treatment in patients with RAS mutant, microsatellite stable, metastatic colorectal cancer: study protocol of a randomized, open-label, multicentric study

**DOI:** 10.1186/s12885-023-11139-z

**Published:** 2023-07-18

**Authors:** Xuefeng Fang, Chenhan Zhong, Shanshan Weng, Hanguang Hu, Jian Wang, Qian Xiao, Jianwei Wang, Lifeng Sun, Dong Xu, Xiujun Liao, Caixia Dong, Suzhan Zhang, Jun Li, Kefeng Ding, Ying Yuan

**Affiliations:** 1 Department of Medical Oncology, Key Laboratory of Cancer Prevention and Intervention, Ministry of Education, The Second Affiliated Hospital, Zhejiang University School of Medicine, Hangzhou, 310009 Zhejiang China; 2 Department of Colorectal Surgery and Oncology, Key Laboratory of Cancer Prevention and Intervention, Ministry of Education, The Second Affiliated Hospital, Zhejiang University School of Medicine, Hangzhou, 310009 Zhejiang China; 3 Cancer Institute, Key Laboratory of Cancer Prevention and Intervention, Ministry of Education, The Second Affiliated Hospital, Zhejiang University School of Medicine, Hangzhou, 310009 Zhejiang China; 4 Cancer Center, Zhejiang University, Hangzhou, 310058 Zhejiang China

**Keywords:** Metastatic colorectal cancer, First line therapy, Sintilimab, CapeOx, Bevacizumab, RAS-mutant

## Abstract

**Background:**

Rat sarcoma viral oncogene homolog (RAS) gene mutation is a common molecular event in colorectal cancer (CRC). The prognosis of mCRC (metastatic colorectal cancer) patients with RAS mutation is poor and capecitabine and oxaliplatin (CapeOx) plus bevacizumab has shown to be one of the standard therapeutic regimens as first line for these patients with objective response rate (ORR) of ~ 50% and median progression-free survival (mPFS) of 8–9 months. Immunotherapy, especially anti-programmed death 1 (PD-1) monoclonal antibody has demonstrated ground-breaking results in deficient mismatch repair (dMMR) / microsatellite instability-high (MSI-H) mCRC patients. However, the response rate of in microsatellite stable (MSS) patients is extremely low. In addition, preclinical studies have demonstrated that anti**-**Vascular endothelial growth factor (VEGF) agents, such as bevacizumab, can induce tumor vascular normalization and enhance antitumor immunity. Previous study indicated the combination of chemotherapy, anti-VEGF agents (bevacizumab) with immune checkpoint inhibitors may have promising clinical activity in RAS mutant, MSS refractory mCRC patients. Based on these evidences, we will explore the combination of CapeOx with bevacizumab and sintilimab (anti-PD-1 monoclonal antibody) in RAS mutant, MSS mCRC patients as first-line therapy.

**Methods:**

This is a randomized, open-label, multicentric clinical trial. In the sintilimab arm, patients will receive sintilimab in combination with CapeOx and bevacizumab. In the control arm, patients will receive CapeOx and bevacizumab. This trial will recruit 494 patients from 20 centers and randomly (1:1) disseminated into two groups. The primary endpoint is the PFS. The secondary endpoints include overall survival, safety, ORR, and disease control rate.

**Discussion:**

This study may provide new ideas for optimizing oncology treatment planning for RAS mutant, MSS mCRC patients in the first-line set.

**Trial registration:**

This study is short for BBCAPX and has been registered at clinicaltrials.gov registry with identifier NCT05171660.

**Supplementary Information:**

The online version contains supplementary material available at 10.1186/s12885-023-11139-z.

## Background

Colorectal cancer (CRC) is the third most common cancer in worldwide, with an estimated incidence of 1.9 million cases in 2020 [1]. In 1988, Volgelstein et al. have demonstrated a model in which accumulated alterations affecting at least one dominantly acting oncogene and several tumor-suppressor genes are responsible for the development of colorectal tumors. They also mentioned that Rat sarcoma viral oncogene homolog (RAS) gene mutations are often relatively early events in colorectal tumorigenesis [2]. The RAS gene (KRAS and NRAS) is mutated approximately in 50–55% mCRC patients [3].

Many scholars believe that RAS mutations are associated with poor outcome in mCRC [4, 5]. A pool analysis of 1239 patients from five randomized trials in metastatic colorectal cancer by the AIO colorectal cancer study group demonstrated that RAS mutation was associated with inferior progression free survival (PFS) and overall survival (OS) of mCRC patients compared with patients with non-mutant tumor [6].Current clinical guidelines recommend chemotherapy combined with bevacizumab (bev) as the standard first-line treatment for mCRC with RAS gene mutation, with an objective response rate (ORR) of ~ 50% and a median mPFS of 8–9 months. It remains a big challenge to improve the prognosis of mCRC patients with RAS mutation.

Cancer immunotherapy aims to enhance the natural capability of the immune system to fight cancer cells and has already become one of the pillars of cancer treatment in advanced stages [7]. Immune checkpoint inhibitors (ICI) are monoclonal antibodies that block these pathways by binding to PD-1/L1 or CTLA-4 and enhance the immune response against cancer cells [7] and have demonstrated ground-breaking results in several cancers [8].

The Food and Drug Administration (FDA) approved pembrolizumab for the treatment of patients with unresectable or metastatic microsatellite instability-high (MSI-H) / deficient mismatch repair (dMMR) colorectal cancer with no prior systemic treatment [9]. However, microsatellite stable (MSS) patients account for 95% of patients with metastatic colorectal cancer [10–12]. The response rate of anti PD-1 monoclonal antibodies in these patients is extremely low. Thus, it is urgent to improve the response of immunotherapy for MSS mCRC patients. Previous studies have indicated that the combination of PD-1 with chemotherapy or anti-angiogenesis can increase its response rate to a certain extent. In a phase II study of pembrolizumab in combination with mFOLFOX6 for patients with advanced colorectal cancer, a total of 30 CRC (3 with dMMR, 22 with proficient-MMR (pMMR) and 5 with no available data) were included, 1 complete response (CR), 15 partial response (PR) (CR + PR = 53%), and 14 stable disease (SD), with 100% disease control rate (DCR) at 8 weeks. Clinical activity was seen in these patients with pMMR [13]. MEDITREME study enrolled 57 patients with RAS mutation and MSS to receive mFOLFOX6 in combination with durvalumab and tremelimumab, and indicated that the ORR and 12-month PFS were 62.5% and 50% respectively [14].

Vascular endothelial growth factor (VEGF)-targeting agents, such as bevacizumab, play an essential role in this process as the blood vessels could present an obstacle to extravasation of immune cells in the interstitial space [15]. VEGF inhibition by bevacizumab can also restore the function and enhance the infiltration of effector T cells, decrease the number of immunosuppressive Tregs, tumor associated macrophages (TAMs), and mast cells, and inhibit the accumulation and immunosuppressive activity of myeloid-derived suppressor cells (MDSCs). Therefore, anti-vascular combined with immunotherapy is an important strategy for the treatment of several advanced cancers.

In a phase Ib trial named REGONIVO (EPOC1603), a total of 25 patients with heavy-treated colorectal cancer were enrolled and regorafenib plus nivolumab were administrated. All patients had received ≥ 2 previous lines of chemotherapy. One patient had MSI-H colorectal cancer, whereas the remaining CRC patients had MSS or pMMR tumors. Objective tumor response was 36%. Median PFS was 7.9 months. Thus, the combination of regorafenib plus nivolumab had encouraging antitumor activity in patients with MSS colorectal cancer, which warrants additional investigations in larger cohorts or randomized trials [16].

In addition, preclinical studies show chemotherapy can potentiate antitumor immunity. In CRC mouse model, oxaliplatin (OXP) can induce anticancer immune response [17]. MDSC may contribute to the immune tolerance in several cancers. In vivo, the treatment of tumor-bearing mice with fluorouracil (5-FU) led to a major decrease of MDSC in the spleens and tumor beds, which suggest that the antitumor effect of 5-FU is mediated, at least in part, by its selective cytotoxic action on MDSC [18].

Sintilimab, a highly selective monoclonal IgG4 antibody against PD-1, which works by blocking the association between PD-1 and its ligands, has been approved by the National Medical Products Administration (NMPA) of China to treat relapsed or refractory classical Hodgkin lymphoma in patients [19, 20]. Compared with nivolumab and pembrolizumab, sintilimab has a similar anti-tumor effect, a better safety profile, and obvious economic advantages in some tumors [19]. In addition, to evaluate the efficacy and safety of sintilimab combined with furaquitinib in the treatment of MSS mCRC, 44 patients who had received ≥ 2-line treatment were enrolled and the results showed the ORR was 22.7%. The median follow-up time was 8.3 months. The median PFS in furaquitinib 5 mg intermittent treatment group and 3 mg continuous treatment group were 6.8 months and 4.3 months, respectively. Treatment related adverse reactions were tolerable. The study is still ongoing. The data showed that sindilimab combined with fruquintinib showed good efficacy and safety tolerance after the failure of standard treatment in MSS patients with mCRC.

Based on these data, there is sufficient evidence to explore the combination of CapeOx with immunotherapy and antiangiogenetic inhibitors in RAS mutant, MSS mCRC patients.

## Methods

### Protocol overview

**Fig. 1:**
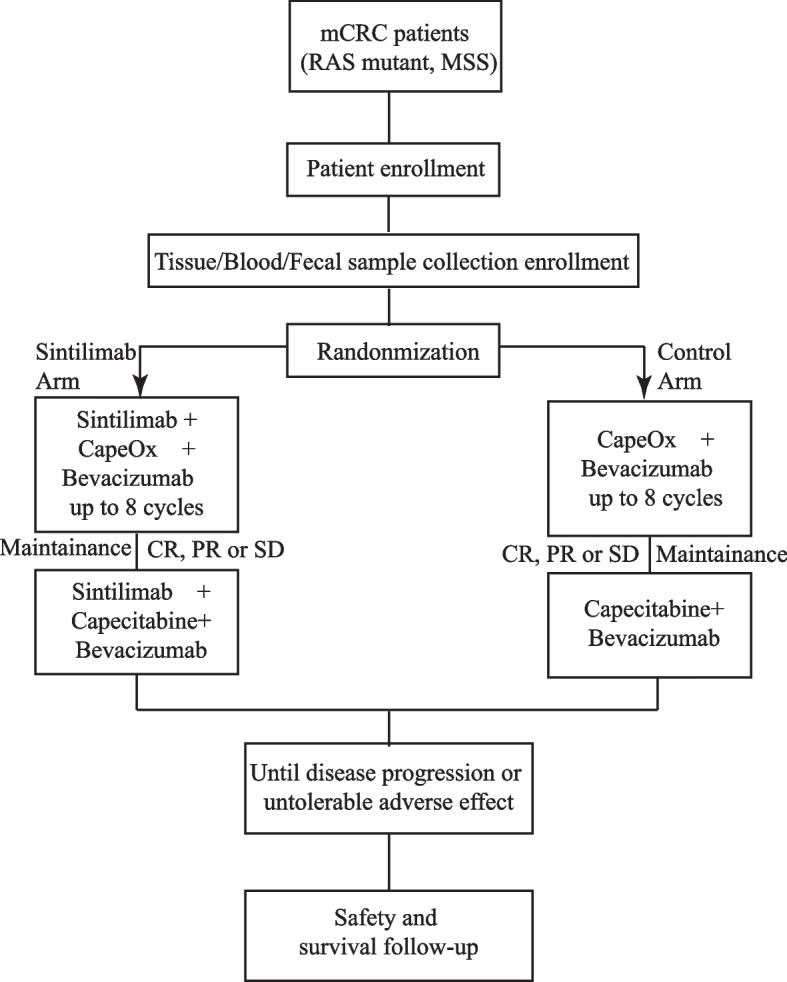
Consort Diagram: Flow of the participants throughout the study

This is a randomized, open-label, multicentric clinical trial to evaluate the effect and safety of sintilimab in combination with CapeOx and bevacizumab as first-line treatment in RAS mutant, MSS mCRC patients when compared with that of CapeOx and bevacizumab. The participated centers can be found in supplementary file 1. The full version of the protocol can be found on the website(https://clinicaltrials.gov/ct2/show/NCT05171660). Patients with mCRC who are planned to participate in this clinical trial will receive study screening within 28 days prior to initiation of the treatment. At screening, every patient must have RAS/BRAF/MSS known status of their primary or metastatic site. Eligible patients will be enrolled and 1:1 randomly allocated to sintilimab and control arms using a web-based system named RTSM Master (https://www.anjusoftware.com/eclinical/rtsm-master/) by the principal investigator. In the sintilimab arm, patients will be administrated with sintilimab combination with CapeOx and bevacizumab for up to 8 cycles (called induction therapy) and in the control arm, patients will be administrated with CapeOx and bevacizumab for up to 8 cycles. In both groups, patients with CR, PR or SD will receive maintenance therapy with sintilimab, capecitabine and bevacizumab (sintilimab arm) or capecitabine and bevacizumab (control arm) every 3 weeks until disease progression, unacceptable toxicity, or patient / physician decision. Sintilimab will be administered intravenously at dose of 200 mg every 3 weeks. Bevacizumab will be administered intravenously at dose of 7.5 mg/kg every 3 weeks. CapeOx consisted of an intravenous infusion of oxaliplatin 130 mg/m^2^ will be given on day 1, followed by oral capecitabine 1000 mg/m^2^ twice a day from day 1 to day 14 of a 3-week cycle. In the control arm, patients will be administrated with CapeOx and bevacizumab, which is the same as in the sintilimab arm (Fig. 1).

During the treatment protocol, patients will be monitored for safety using adverse event (AE) assessments, including vital signs, physical findings, and clinical laboratory test results. The efficacy of the treatment will be evaluated by the investigator using the RECIST 1.1 criteria every two cycles during induction therapy, and then every three cycles during maintenance therapy for up to two years. After discontinuation of treatment, safety assessments will be conducted 30 days after the last drug administration or until the initiation of other anti-cancer therapy. Thereafter, patients will be followed up for disease progression, unless it has already occurred, as well as for serious adverse events, anti-cancer therapy, and survival. The follow-up will continue for up to two years. Before starting the treatment, formalin-fixed and paraffin-embedded (FFPE) tumor samples will be collected. Blood and / or fecal samples will be collected at baseline, before cycle 3, 5, 7, at the end of chemotherapy and at disease progression.

### Subjects

The inclusion and exclusion criteria are listed as follows.

Inclusion criteria:

- 1) Participants must be male or female and be between the ages of 18 and 75 years old.
- 2) Participants must have metastatic colorectal adenocarcinoma that has been confirmed by histology. The metastases cannot be resected and must have been evaluated by a multidisciplinary group.
- 3) Participants must have a RAS mutation, be BRAF wild type, and have MSS.
- 4) Participants must have an ECOG score of 0 to 1.
- 5) Participants must have a life expectancy of more than 12 weeks.
- 6) Participants must have normal hematological examination results, including an absolute neutrophil count (ANC) greater than 1.5 × 10^9^/L, hemoglobin greater than 8 g/dL, and platelet count greater than 80–100 × 10^9^/L (according to the normal values of the clinical trial center).
- 7) Participants must have a prothrombin time (PT) less than 1.5 times the upper limit of the normal value and an activated partial thromboplastin time (APTT) less than 1.5 times the upper limit of the normal value.
- 8) Participants must have normal laboratory examination results, including a serum creatinine level less than or equal to 1.5 times the upper limit of the normal reference range or a creatinine clearance greater than 50 ml/min.
- 9) For participants without liver metastasis, their alanine transaminase (ALT) or aspartate transaminase (AST) levels must be less than or equal to 2.5 times the upper limit of the normal value reference range, and their serum total bilirubin levels must be less than 1.5 times the upper limit of the normal value reference range.
- 10) Women participants of childbearing age must agree to use adequate contraception during treatment with the study drug.
- 11) Participants should sign the consent.
- 12) Patients should have measurable sites according to RECIST 1.1. It should be noted that tumor lesions located in previous radiotherapy areas were considered measurable if they showed progression.

Exclusion criteria:

- 1) Active autoimmune disease requiring systemic treatment within the past 2 years.
- 2) Diagnosis of immunodeficiency or receipt of systemic steroid therapy or any other form of immunosuppressive therapy within 7 days prior to the first dose, unless approved by the sponsor for the use of a physiological dose of corticosteroids.
- 3) Known progression or requirement for active treatment of any other malignancies, except for basal cell carcinoma, cutaneous squamous cell carcinoma, or cervical carcinoma in situ that have undergone radical treatment.
- 4) Known existence of active central nervous system (CNS) metastasis and/or cancerous meningitis;
- 5) Active infections requiring systemic treatment.
- 6) Any conditions that may put patients treated with the study drug at risk, interfere with the evaluation of study drug or subject safety, or impact the interpretation of study results. Additionally, the investigator must believe that participation in the study is in the best interests of the subjects.
- 7) Known mental or substance abuse disorders that may impact compliance with test requirements.
- 8) Female subjects who are pregnant or lactating, or who are expected to become pregnant during the planned trial period, or male subjects whose spouse is willing to become pregnant during the trial period.
- 9) A history of human immunodeficiency virus (HIV) infection (HIV 1/2 antibody);
- 10) Active hepatitis B or C.
- 11) Administration of live vaccines within 30 days of the start date of the study treatment plan.

### Data collection, follow up and management

Subjects’ medical records will be reviewed by research staff on a continual basis every 3 weeks while enrolled, and the database will be created and included the information listed as follows:

• Demographic and clinical data: gender, the age of diagnosis, height, weight, date of sample collection, ECOG score of each subject will be recorded.
• Oncology data: tumor type, histology, the location of primary lesion, stage, as well as the location and the size of metastases will be documented.
• Laboratory assessments include standard tests obtained as part of routine care, such as biochemistry (renal and hepatic function), complete blood count, tumor markers, coagulation, myocardial enzyme spectrum, and thyroid function tests.
• All adverse events and toxicities experienced by enrolled subjects will be recorded, along with their respective dates, and updated continuously for each subject when they seek medical attention. Toxicities may be discovered by the study team through medical record review, notification from a participating subject or their provider, or via surveys administered every six weeks to enrolled subjects.
• Oncologic outcomes: The first radiological assessment will take place 6 weeks (+ 7 days) after the first day of the first cycle treatment. After that, these assessments will be made every 2 cycles (~ 6 weeks) during the induction therapy until the patients completed up to 8 cycles or disease progression. After 8 cycles of induction therapy, the patients with CR, PR or SD will be given maintenance treatment, and assessment will be made every 3 cycles (~ 9 weeks) until disease progression. For patients with bone metastases and / or brain metastases, if there is no aggravation of clinical symptoms during the study, there is no need to do the evaluate of those metastases each time; if the clinical symptoms are aggravated, the study team should assess the metastasis in time. For patients suspected of disease progression before the next scheduled tumor evaluation, an unplanned tumor evaluation should be performed. The post-treatment follow-up visits will occur every 3 months (± 14 days) for up to 2 years. To evaluate progression-free survival (PFS) and response rate, the study team will review progress notes from the treatment team and radiological assessments to confirm disease status, tumor response, and track subsequent changes in therapy. The team will monitor response rate, PFS, and overall survival (OS).
• Data management: The ShangHai Ashermed healthcare communications co.,Ltd is responsible for data management using an electronic data collection system (EDC). The EDC will automatically record inspection marks for all role operations, including data preservation, modification, deletion, proofreading, review, freezing, electronic signature, and locking.

### Study endpoints

The present trial will determine if adding sintilimab to the first line therapy with CapeOx and bevacizumab is efficient in terms of PFS in RAS mutant, MSS mCRC patients.

*Primary endpoint* is PFS, which defined as the time between the beginning of the study-drug administration and physician-determined disease progression or death.

*Secondary endpoints* are the following: (i) Overall Survival (OS), defined as the duration from the commencement of study drug administration until the date of death from any cause. (ii) Overall Response Rate (ORR), defined as the best response recorded in the intent-to-treat (ITT) population in accordance with the Response Evaluation Criteria in Solid Tumors (RECIST) version 1.1. Scale scores can be obtained for multi-item scales. (iii) Safety assessment of the combination treatment with CapeOx and bevacizumab plus sintilimab, graded according to the National Cancer Institute (NCI) Common Terminology Criteria for Adverse Events (CTCAE) version 5.0.

The collateral study will also explore new biomarkers for predicting immunotherapy efficacy and get better guidance for single-drug and combination therapy with immunotherapy. In order to identify new biomarkers, we will collect biological samples (tumor tissues, blood and fecal samples). FFPE tumor samples will be collected before starting fist-line therapy. Blood and / or fecal samples will be collected at different points: at baseline, prior to cycle 3, 5, 7, at the end of chemotherapy and at disease progression. We will perform comprehensive analysis for exploratory biomarkers including PD-L1 expression level, TMB level, Whole exome sequencing (WES), Nanostring panel RNA sequencing, cell infiltration analysis, tumor immune microenvironment (TIME) signature analysis with baseline biopsy or operative specimens. Moreover, the blood samples will be sent for whole genome sequencing to detect the circulating tumor DNA (ctDNA). As for the fecal samples, the Whole Genome Shotgun and Liquid chromatography-mass spectrometry (LCMS) will be done to explore the microbiome and metabonomics of RAS MT MSS CRC.

### Statistical analysis and sample size

The sample size was determined by the PFS of 12 months required to show superiority of sintilimab arm to control arm (12 months in sintilimab arm VS 9 months in control arm). 494 patients will be enrolled to achieve 380 PFS events, which will allow an 80% power to detect a hazard ratio of 0.65 comparing sintilimab arm and control arm with one-sided α level of 0.025, allowing a 10% attrition rate. SAS will be used for all statistical analyses. The Kaplan–Meier method will be used for the analyses of PFS. The differences in ORR between two groups will be assessed with the stratified Miettinen and Nurminen method.

### Confidentiality

The measures to protect confidentiality can be summarized as follows: Only a unique study number will be used to identify patients in the electronic case report form (eCRF) or other documents that will be submitted to the sponsor. Patient names and any other personal information that is not necessary will not be entered in the eCRF. The patients' rights will be outlined in the informed consent form (ICF), and insurance coverage will be provided for each patient. The principal investigator will have access to the final trial dataset, and no media coverage will be permitted during the study period.

## Discussion

At present, chemotherapy combined with bevacizumab could elongate the survival and has been considered as the standard first-line treatment for mCRC patients with RAS gene mutation. However, the prognosis of RAS mutant mCRC patients are still poor [4, 5].

Immunotherapy, particularly ICIs, has achieved observable and durable responses in some solid tumors, providing a new approach for cancer treatment [21–23]. In a phase III clinical trial (NCT02563002), KEYNOTE-177, pembrolizumab was utilized as a first-line therapy in patients with dMMR/MSI-H mCRC and has achieved significantly elongated PFS. Besides, the ORR was 45.1% in the pembrolizumab group compared with 33.1% in the chemotherapy group [24]. However, the response rate of ICIs in patients with MSS mCRC is very low, partly owing to its high immunosuppression, low tumor mutation burden (TMB) and low expression of neoantigens. In addition, several studies have demonstrated that RAS mutations could regulate the tumor microenvironment in various cancer types [25]. Moreover, Lal et al. reported that the reduced infiltration of cytotoxic T cells and downregulation of the interferon gamma (IFN-γ) pathway was seen in KRAS-mutant CRC [26], and Liao et al. found that KRAS mutations induced an immune-suppressive profile by inhibiting interferon regulatory factor 2 (IRF2) expression and promoting the migration of MDSCs [27]. Moreover, Park et al. indicated G12D/V KRAS mutation was consistently associated with less tumor infiltrating lymphocytes (TIL) infiltration and shorter recurrence free survival (RFS) in stage III CRC patients treated with adjuvant FOLFOX therapy [4].

VEGF-targeting agents, such as bevacizumab, can increase the immunogenicity as studied with preclinical tumor models [28]. As is shown in multiple studies, the inhibition of VEGF reduced immunosuppressive cell population, increased TILs and improved T-cell function [29] thus enhancing anti-tumor activity. Results of another study with MSS mCRC patients suggested that VEGF inhibitors can upregulate immune checkpoint responses involving the thymocyte selection-associated high mobility group box protein (TOX) transcription factor, which can mediate CD8 + T-cell exhaustion [30].

In addition, preclinical studies show that chemotherapy can potentiate antitumor immunity. 5-FU administration can enhance antitumor immune responses by reducing Tregs and MDSCs number and recruiting dendritic cell (DC) infiltration of tumors [31, 32] in CRC mouse model. Subcutaneous injection of OXP-treated CT26 cells induced an anticancer immune response. CT26 tumors implanted in immunocompetent mice responded to OXP treatment in vivo. OXP elicits immunogenic cell death in several rodent models of colon cancer, and this effect determines its therapeutic efficacy in CRC patients [17]. In CRC patients, it has also been validated that 5-FU can increase the expression of PD-L1 on CRC cells and improve the response rate with ICI intervention [33, 34]. Furthermore, a large number of clinical studies have also shown that ICI interventions in combination with chemotherapeutic agents can improve outcomes for many types of cancers [35–37].

Recently, several clinical studies have released results of the use of chemotherapy combination of bevacizumab and PD-L1 / PD-1 in metastatic CRC patients, which demonstrated that the combination is a promising strategy, indorsing clinically meaningful and durable benefit for MSS CRC patients.

A phase II clinical trial of bevacizumab plus capecitabine and atezolizumab (atezo) in MSS metastatic refractory colorectal cancer reported promising clinical activity [38]. An ORR of 8.54% and median PFS of 4.4 months were recorded in the triplet arm compared to 4.35% and 3.3 months in the bevacizumab plus capecitabine arm. Overall, no unexpected treatment-related AEs were reported, and no treatment-related AEs led to treatment discontinuation or death.

From these preclinical and clinical results, the combination of chemotherapy with ICIs and bevacizumab may be worthy of investigation in patients with RAS mutant and MSS mCRC patients. Up to date, a prospective, open-label, multicentric phase II trial where patients with mCRC RAS / BRAF mutant, in first line will receive nivolumab in combination with FOLFOXIRI / bevacizumab is ongoing [39]. Another study evaluated the efficacy of atezolizumab in combination with bevacizumab and FOLFOXIRI (AtezoTRIBE study) in mCRC patients with unslected MSI status [40] is also ongoing. According to the recent results released in European Society of Medical Oncology (ESMO) congress 2021, a significant advantage by the addition of atezo was observed in PFS (13.1 months of arm B FOLFOXIRI / bev / atezo vs 11.5 months of arm A FOLFOXIRI / bev, HR 0.69, 80% CI 0.56–0.85, *p* = 0.012), but not in ORR (59% vs 64%, *p* = 0.412). Significant interaction effect between MMR status and treatment arm was also found (*p* = 0.010). In the pMMR subgroup (*N* = 199, arm A/B: 67/132), 147 (arm A/B: 54/93) PFS events were collected. Significantly longer PFS was reported in arm B (12.9 months vs 11.4 months, HR 0.78, 80% CI 0.62–0.97, *p* = 0.071) [41]. In this study, the primary endpoint was met, and the addition of atezo to FOLFOXIRI / bev prolongs PFS of mCRC patients. While the use of the combination of CapeOx, bevacizumab and anti-PD-1 monoclonal antibody in RAS mutant metastatic CRC patients as first-line treatment is still lacking.

Several studies have demonstrated that sintilimab is well tolerated and has similar anti-tumor effect when compared with nivolumab and pembrolizumab in solid tumors [42]. Up to now, there is limited data about the use of sintilimab in colorectal cancer. An open-label, phase II, single-arm study indicated that sintilimab is quite effective and may be an alternative for dMMR/MSI-H locally advanced rectal cancer patients [43]. In addition, Guo et al. has released the preliminary results of a phase 1b study of fruquintinib plus sintilimab in mCRC patients (MSI status unselected) after failure to the standard therapies. The overall ORR was 22.7% [44]. The combination showed promising efficacy and manageable safety profile. However, there is no data reported in MSS mCRC patients using sintilimab as first-line therapy. Also, to assess the antitumor activity and safety of sintilimab combined with CapeOx and bevacizumab for patients with RAS-mutant MSS mCRC in the first line therapy, we conducted a single arm, phase II study. Up to now, thirteen mCRC patients were enrolled and received at least two cycles of treatment. Notably, one of the 13 patients got complete response and 11 patients got partial responses (data unpublished).

In conclusion, we assume that there is sufficient evidence to support the combination of treatment with CapeOx, anti-VEGF agents (bevacizumab), and sintilimab in RAS mutant, MSS mCRC patients. in the first-line setting. Thus, we designed this clinical study.

## Supplementary Information

**Additional file 1: Supplementary file 1. **A list of participating centers of BBCAPX trial.

## Data Availability

Results will be shown via presentations at international meetings and via publications in peer-reviewed journals. The corresponding author could provide data generated or analyzed during the current study via reasonable request.

## Abbreviations

RAS: Rat sarcoma
BRAF: V-Raf murine sarcoma viral oncogene homolog B1
CapeOx: Capecitabine, oxaliplatin
PD-1: Programmed death cell protein 1
dMMR: Deficient mismatch repair
MSI-H: Microsatellite instability-high
TAM: Tumor associated macrophages
MDSC: Myeloid-derived suppressor cells
VEGF: Vascular endothelial growth factor
OS: Overall survival
ORR: Overall response rate
DCR: Disease control rate
CRC: Colorectal cancer
EGFR: Epidermal growth factor receptor
FOLFOXIRI: 5-Fluorouracil, oxaliplatin, irinotecan
FOLFOX: 5- Fluorouracil, oxaliplatin
RFS: Recurrence free survival
ICI: Immune checkpoint inhibitor
CTLA-4: Cytotoxic T-lymphocyte-associated protein 4
PFS: Progression free survival
AE: Adverse event
ECOG-PS: Eastern Cooperative Oncology Group – performance status
NCI CTCAE: National Cancer Institute Common Terminology criteria for adverse events
CR: Complete response
PR: Partial response
SD: Stable disease
TIL: Tumor infiltrating lymphocytes
ESMO: European Society of Medical Oncology
HR: Hazard ratio
ALT: Alanine transaminase
AST: Aspartate transaminase
OXP: Oxaliplatin
ITT: Intent-to-treat
ctDNA: circulating tumor DNA
LC-MS: Liquid chromatography-mass spectrometry
eCRF: Electronic case report form
ICF: Informed consent form
