# Supplementary material for: Sintilimab plus bevacizumab and CapeOx (BBCAPX) on first-line treatment in patients with RAS mutant, microsatellite stable, metastatic colorectal cancer: study protocol of a randomized, open-label, multicentric study
Source: BMC Cancer. 2023 Jul 18;23:676. doi: 10.1186/s12885-023-11139-z (PMC10354966; doi:10.1186/s12885-023-11139-z)
Supplement: Supplementary file 1 — Additional file 1: Supplementary file 1. A list of participating centers of BBCAPX trial. [file 12885_2023_11139_MOESM1_ESM.docx]

Supplementary file 1 of BBCAPX trial

**List of participating centers:**
1. Jiangsu Cancer Hospital,Jiangsu,China

2. The First Affiliated Hospital of Soochow University, Jiangsu, China

3. The First Affiliated Hospital of Nanchang University, Jiangsu, China

4. Huzhou Central Hospital, Zhejiang, China

5. Zhebei Mingzhou Hospital, Zhejiang, China

6. Taizhou Hospital of Zhejiang, China

7. The First Hospital of Jiaxing, Zhejiang,China

8. The Second Hospital of Jiaxing, Zhejiang,China

9. Lishui People's Hospital Zhejiang, China

10. Jiashan County People's Hospital, Zhejiang, China

11. Jinhua Municipal Central Hospital Group, Zhejiang, China

12. Jiangsu Province Hospital, Jiangsu, China

13. The First Affiliated Hospital of Wenzhou Medical University, Zhejiang, China

14. Dongyang People’s Hospital, Zhejiang, China

15. TCM Hospital Changxing, Zhejiang, China

16. West China Hospital, Sichuan University, Sichuan, China

17. Sir Run Run Shaw Hospital, Zhejiang University, School of Medicine, Zhejiang, China

18. Ningbo Medical Center Lihuili Hospital, Zhejiang, China

19. The Affiliated Hospital of Medical School, Ningbo University, Zhejiang, China

20. Shandong Cancer Hospital, Shandong, China
